# Sorption and Micro-Scale Strength Properties of Coals Susceptible to Outburst Caused by Changes in Degree of Coalification

**DOI:** 10.3390/ma14195807

**Published:** 2021-10-04

**Authors:** Katarzyna Godyń, Barbara Dutka

**Affiliations:** Strata Mechanics Research Institute of the Polish Academy of Sciences, Reymonta 27, 30-059 Kraków, Poland; biuro12@imgpan.pl

**Keywords:** coal, methane, sorption capacity, effective diffusion coefficient, microhardness, coalification degree, outburst hazard

## Abstract

Coals from the south-western part of the Upper Silesian Coal Basin have a strong outburst susceptibility. The objective of this study was to identify the influence of coalification degree on methane sorption and micro scale strength properties of 24 coals from Jastrzębie Zdrój. Coal samples showed a reflectance R_o_ between 0.98 and 1.25%. Sorption measurements were carried out by gravimetric method. Sorption capacities were determined at mean deposit temperature of 35 °C. Using the unipore model and solution of Fick’s second law, the effective diffusion coefficients of methane in the studied coals were obtained. The Vickers method was used to study the microhardness and the modulus of elasticity. It has been shown that the increase in the coalification degree reduces the sorption capacity of coal and also reduces the rate of methane emission. Coals the most susceptible to outbursts, were the most brittle. With the increase in R_o_, the methane seam pressure *p* increased as well as desorbable methane content *DMC*, both due to the reduction in the sorption capacity of coal. The increased *dp* index is a warning sign indicating an increased total methane content of coal seam, an increased seam pressure or an alternation of coal structure.

## 1. Introduction

Coal is still the basic energy resource in Poland [1]. It is expected that in the coming years, the domestic power industry will be based mainly on coal [2]. Combined energy management, including the simultaneous production of electricity and heat, is currently the optimal way of coal management. An alternative way of using coal is the production of coke, of which Poland is a significant European producer. Various products of modern organic chemistry can be obtained from coal [3]. The process of coal hydrogenation allows for the production of synthetic fuels [4]. Underground gasification of coal deposits enables the extraction of methane (ecological fuel) and such gases as hydrogen and carbon monoxide. Despite the above, actions taken to protect the climate result in a reduction in the consumption of hard coal in the entire European Union [5].

Coalbed methane (CBM), present in the form of gas adsorbed within the microporous coal matrix and, in a small part, as a free gas is an unconventional natural gas resource [6]. Mining activity in coal seams saturated with methane contributes to gas-geodynamic phenomena, with outbursts posing the greatest risk. Coal and methane outburst is a result of many factors acting together, with the gaseous and mechanical properties of coal being decisive for the occurrence of this phenomenon. It is a dynamic process, in which the kinetic properties of gas sorption/desorption are responsible for sustaining the rock material ejected into the excavation, and therefore also for the extent of the disaster. Complicated geological and mining conditions as well as increasing depth of extraction contribute to the increased risk of coal and methane outbursts [7,8].

The occurrence of uncontrolled gas-geodynamic phenomena in the seams of the Upper Silesian Coal Basin (USCB) still poses a real threat, particularly in the south-western part, where coal seams with medium degree of coalification are located. Two serious coal and methane outbursts with tragic consequences occurred: in 2002, in the Pniówek Mine and in 2005, in the Zofiówka Mine [9]. Another outburst occurred in 2012 in the Budryk mine, seam 358/1 at the level of 1050 m [10]. Outbursts still occur today, but at a lower scale and rarely seen in the news.

In Polish mining, the most important parameters describing a coal-methane system in situ are total methane content *Mn* and desorption intensity index *dp*. The total methane content is defined as the volume of methane included in undisturbed seam, while the desorption intensity index specifies the quantity of methane released from a coal sample of 0.5 ÷ 1.0 mm grain fraction between the second and the fourth minute after desorption was initiated. In mining practice, the mechanical properties of coal are represented by a single parameter—firmness *f* [11]. According to the regulations of Geological and Mining Law [12], the threshold values of the aforementioned parameters enable classification of coal seams or their parts to the category of susceptible to outbursts: (i) if the total methane content exceeds 8 m^3^/Mg and coal firmness is lower than the threshold value of 0.3, or (ii) if the total methane content does not exceed 8 m^3^/Mg, coal firmness is at least 0.3, and methane desorption intensity is greater than 1.2 kPa [13]. Coal seams or their parts are considered as threatened by outbursts if coal and methane outburst or a sudden methane release occurred or if specific symptoms were identified (e.g., cutting blowouts, drills becoming stuck or pushed out, coalface cracking noises, decreased firmness, etc.).

It is commonly known that the most of the outbursts occur in areas of tectonic dislocations, in structurally altered coal [14,15,16]. Such coal is “unstable” as a result of its decreased firmness [8]. Therefore, researchers are trying to identify the important properties of coal that contribute to the increased outbursts hazard [17,18,19,20,21]. All the properties are closely related to the coalification degree of the organic matter [22,23,24,25,26].

Many parameters supporting the assessment of outburst risk are determined under laboratory conditions. Gas parameters include the sorption capacity of coal *a* and the effective diffusion coefficient *D_e_*. Sorption capacity determines the seam ability to accumulate gas, while the effective diffusion coefficient is decisive for the rate of gas release from the seam. Both parameters are significant, as both the *Mn* and *dp* measured in situ are directly derived from the values of *a* and *D_e_*, respectively. Micro scale laboratory test is performed in order to determine mechanical parameter called Vickers microhardness *H_v_*. The analysis of the aforementioned parameters constitutes a significant contribution to the experts’ opinions deciding on the level of the outbursts risk in mines. Understanding the sorption properties of coal as well as the identification of strength properties is of practical importance in many aspects of development and application of CBM. It is also relevant in the control and prevention of coal and methane outbursts in active mines.

The objective of this study was to investigate the effect of coalification degree on the sorption and micro-strength properties of 24 coals susceptible to outbursts. The research was supplemented with the assessment of the impact of coalification degree on the parameters determining the risk of coal and methane outburst. The article contributes significantly to the identification of the inherent properties of coals that are susceptible to outbursts in order to prevent outbursts in active coal mines.

## 2. Properties Examined in the Study

### 2.1. Degree of Coalification

Coal is a biogenic sedimentary rock (a so-called caustobiolite), formed mainly from various plant remains that underwent coalification as a result of various transformations and varied conditions [27]. Such transformations result in an increase of the elemental C content in the sediment, accompanied by a reduction in the hydrogen and oxygen content (Schűrmann’s rule) [28]. At the same time, intensified coalification leads to a decrease in volatile content (Hilt’s law) [28,29]. Depending on the degree of coalification (coal rank), coal contains between 75% and 96% of elemental C within its structure. Evaluation of the coal rank is based on the result of vitrinite reflectance (R_o_) measurements, which is a numerical expression of the ratio of the intensity of reflected light to the intensity of incident light radiated perpendicular to the polished sample (section) surface. R_o_ is a sensitive coalification parameter, and it is commonly considered to be an indicator of the coalification scale in solid fuels [27]. The degree of coalification is a parameter determining several coal properties. A higher degree of coalification results in a higher gloss, changes to microscopic properties (e.g., colour or structure), along with changes to strength properties [23,30], accumulation properties [24], etc. Therefore, assessment of the degree of coalification is the key and the starting parameter required for further studies of coal.

### 2.2. Sorption Capacity

The assessment of susceptibility to outburst of coal seams requires information about the quantity of gas present in the coal. Methane in coal seams is present mainly as adsorbed gas in the microporous coal matrix, and to a small degree as a free gas in the system formed by macropores, cracks and fractures. Adsorbed methane constitutes more than 95% of methane present in the seams [31], therefore it determines their total methane content (*Mn*) [6,32]. The sorption capacity *a* is a quantitative parameter indicating the amount of methane absorbed in a mass unit of coal under sorption equilibrium conditions, constant temperature and gas pressure. In mining practice, the sorption capacity of coal under methane pressure of 0.1 MPa and at 25 °C (*a*_0.1_) is commonly used. By comparing the value of *a*_0.1_ with the total methane content *Mn*, it is possible to infer a potential increase in outburst risk. Special attention should be paid to situations where a high total methane content is accompanied by a relatively low sorption capacity *a*_0.1_. Such situations may indicate an increased coal seam methane pressure *p* and increased desorbable methane content (DMC), which may be released from the coal to the excavation. The most important factors influencing the sorption capacity of coal include pressure, deposit temperature, moisture content, the degree of coalification of the coal substance, the maceral composition and lithostatic loads [33]. Information on the effect of the degree of coalification is required to evaluate changes to the sorption capacity of coal seams with depth within the given mining area [7].

### 2.3. Effective Diffusion Coefficient

Assessment of hazards related to the presence of methane in coal seams is based on an analysis of the coal gas emission rate, which is simply indicated by an underground measurement of the desorption intensity index *dp* [34,35]. As a result, methane sorption or desorption kinetic properties are evaluated, which (as equivalent processes) are dictated by the value of the effective diffusion coefficient $D_{e}$. This coefficient provides important information about the porous structure of coal and the gas transfer mechanisms within a seam [36]. Pressure, temperature [37,38] and moisture content [39] have a significant impact on the value of $D_{e}$. The wide range of variability of methane diffusion coefficient values for coal, between 10^−11^ cm^2^/s and 5 × 10^−7^ cm^2^/s [36] allows coal types to be differentiated in terms of the potential methane and outburst risk. The high value of $D_{e}$ may also be a result of structural changes to coal [40], determining the outburst susceptibility of coals.

### 2.4. Coal Microhardness

There are several methods used to determine coal hardness. One of the most commonly used methods is the Hardgrove Grindability Index (HGI) [28,41]. This method is used to measure the susceptibility of the given material to grinding [42]; the lower the value, the harder the coal. Microscale measurements are used to determine the hardness of individual macerals. The Vickers method is the most frequently used method of determining coal microhardness [23,36]; the Knoop method is also sometimes used for this substance [43]. Microhardness has been used by a number of researchers to examine coal for many years [44,45], including the Upper Silesian Coal Basin [23,30,46,47,48,49,50,51,52] and in other coal basins (e.g., Indian coals [41] or highly volatile, bituminous Kentucky coals [53]). Some researchers use microhardness tests to solve unusual problems—for example, Hower et al. [53] presented an interesting use of Vickers microhardness tests to distinguish almost identical macerals of the vitrinite group. According to many researchers, microhardness is a very good indicator used to detect weathered coal fragments. Most researchers think that oxidation causes increased microhardness [54,55,56,57].

## 3. Materials and Methods

### 3.1. Area of Study

**Figure 1:**
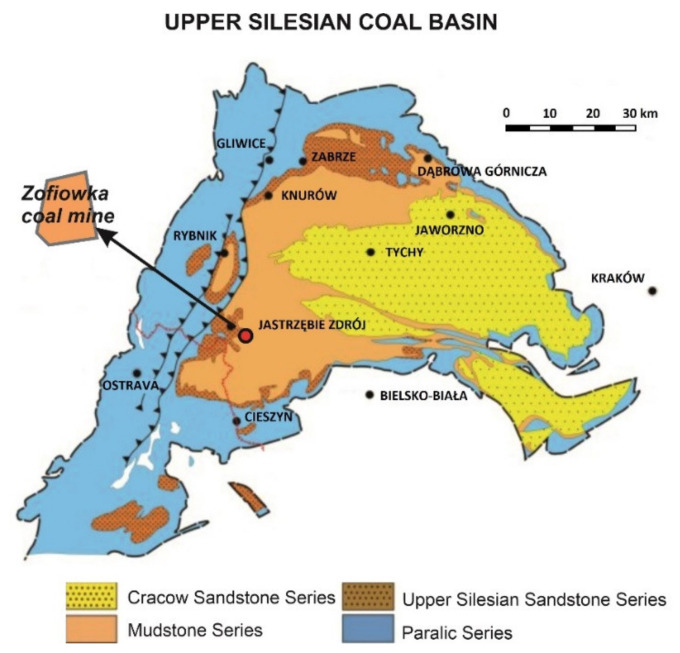
Location of the Zofiówka Mine mining area as the sampling area [62,63].

The area of study is located within the Upper Silesian Coal Basin. This basin was formed during the Varisician orogeny, mainly in the Asturian stage [58]. It is a piedmont subsidence formed as a synclinal basin, filled with coal-bearing formation dating to the Upper Carboniferous period [59]. The Basin is divided into three zones with different tectonic styles: fold, fold-block and disjunctive styles [60]. The area of study is located within a disjunctive tectonics area, which covers the majority of the Upper Silesian Basin. The formation of this area is related to the block structure of the substrate, with faults as its main structural element. The Alpine orogeny had a significant impact on the structure of this area and of the entire Basin, as in this period a large number of faults was reanimated, with their amplitudes increased. The area of study includes the Zofiówka Mine seams available for sampling in 2014. The mine is located in the so-called Zofiówka monocline [61], entirely located within the disjunctive tectonics area (Figure 1). The coal deposits of the Zofiówka consist of multiple seams, down to the depth of 1080 m [62]. 42 seams were located within the deposit, including 5 “Orzeskie”, 30 “Rudzkie” and 7 “Saddle” seams. Numerous hazards were identified in the mine, including methane and rock outbursts.

### 3.2. Coal Samples

The tests described in this paper were performed on 24 coals sampled from various seams in the Zofiówka Mine (Figure 1). Samples collected from side walls were secured in closed containers, followed by crushing and sieving into two grain classes, 0.5–1.0 mm and 0.125–0.16 mm, respectively. Information on the studied Zofiówka Mine seams is presented in Table 1. In terms of stratigraphy, the deposits belong to Westphalian stage (Załęskie strata) and the Namurian stage (Rudzkie and Siodłowe strata) [58].

### 3.3. Characteristics of Coals

The basic characteristics of coals were obtained via technical analysis, which determined the total moisture content *W^t^*, volatile content *V^daf^* and ash content *A^d^* in the coal [30].

### 3.4. Measurement of Coalification Degree

The degree of coalification was determined for each of the samples by measuring the average vitrinite reflectance R_o_ on parts of a meceral from the vitrinite group: colotelinite [64]. Polished grain preparations (sections) were prepared from coal crushed down to 0.5 ÷ 1.0 mm. Reflectance was measured using an Olympus BX50 polarisation microscope, at 400× magnification. The polished preparations were examined under monochromatic light, using oil immersion. The results were obtained using the LUCIA Vitrinite image analysis system [65].

### 3.5. Sorption Studies

**Figure 2:**
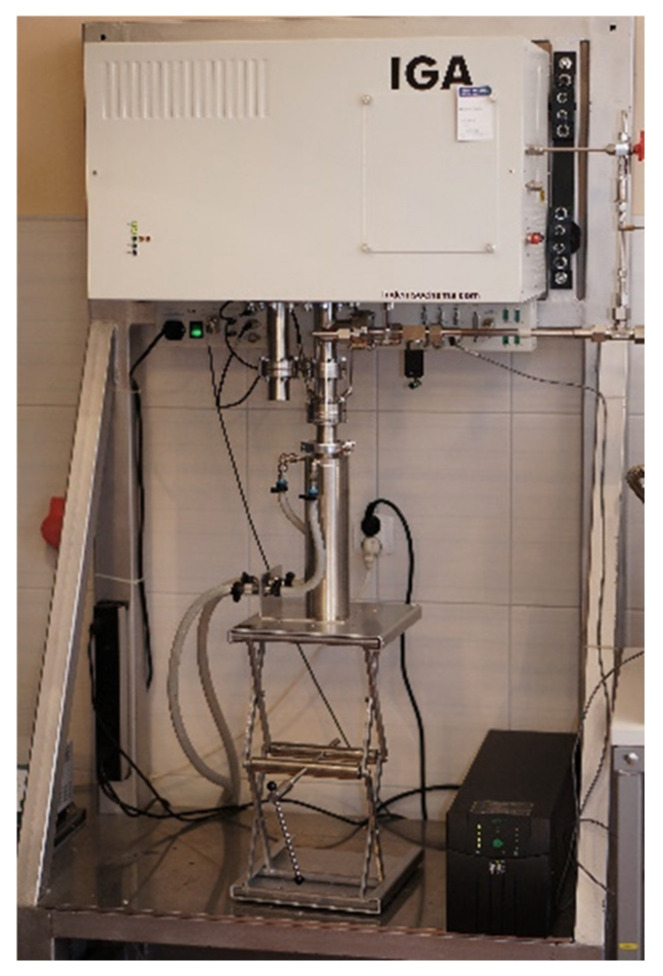
Gravimetric sorption analyser IGA-001: photo of the stand.

The goal of the sorption tests was to determine methane sorption isotherms at the mean deposit temperature (35 °C) and in the methane pressure range from 0 to 1.0 MPa, including kinetic curves. Measurements were performed using the gravimetric method and an IGA-001 sorption analyser by Hiden Isochema (Figure 2). Dry samples of coal in the 0.16 ÷ 0.25 mm class were used in the measurements, degassed at 80 °C for approximately 10 h, down to *p* = 10^−6^ mbar. Sorption isotherms were prepared on the basis of measurements of the quantity of absorbed methane for the following equilibrium pressures: 0.1 MPa (ambient pressure), 0.3 MPa and 1.0 MPa. The Langmuir isotherm model (1) was used to describe methane sorption on the studied coals in the form of [26]:

(1) $$a\left(p\right)\;=a_{m}\frac{p}{p_{L}+p},$$

where:

a—methane absorbed at pressure *p*, cm^3^CH_4_/Mg (*STP*: 25 °C and 0.1 MPa; daf).
p—methane equilibrium pressure, bar.
T—temperature, °C.
$a_{m}$—maximum sorption capacity at *p* approaching ∞, cm^3^CH_4_/Mg (*STP*, daf).
$p_{L}$—Langmuir pressure, MPa.

Constants present in the Langmuir isotherm equation: $a_{m}$ and $p_{L}$ include information on the shape of a hyperbolic isotherm, as well as on the aspects of the sorption process itself. The $a_{m}$ constant defines the maximum sorption capacity of coal at a given temperature, if gas pressure within the system reaches the maximum value and assuming a finite quantity of sorption points (so-called monolayer capacity). The *P_L_* specifies the half-sorption pressure. The lower the *P_L_* value, the stronger the sorption at low pressures.

The analysis of methane sorption kinetic properties at 0.1 MPa enabled estimation of the effective diffusion coefficient *D_e_* for methane in the studied coals. A unipore diffusion model solution (2) for coal grains was used, which has the following form for the sorption process [66,67]:

(2) $$\gamma =\frac{a\left(t\right)}{a_{\infty }}=1-\frac{6}{\pi^{2}\;}\;\sum_{n=1}^{\infty }\left[\frac{1}{n^{2}}exp\left(-\frac{n^{2}\pi^{2}}{R^{2}}\cdot D_{e}t\right)\right],$$

where:

γ—relative quantity of absorbed gas, -.
$a\left(t\right)$—quantity of gas absorbed at the time of t, cm^3^CH_4_/g.
$a_{\infty }$—total quantity of gas absorbed in coal under measurement conditions, cm^3^CH_4_/g.
R—equivalent grain radius for a sample with a specific grain class,
$D_{e}$—effective diffusion coefficient, cm^2^/s.
t—is the time, s.

The formula (3) obtained by Timofeev [66] was used to determine *D_e_*:

(3) $$D_{e}=\frac{0.308\;R_{z}{}^{2}}{\pi^{2}t_{0.5}},$$

where:

$t_{0.5}$—is the half-time required to reach the relative absorbed quantity γ = 0.5, s.

According to conclusions from the study by Busch and Gensterblum [68], the unipore model precisely represents sorption kinetic properties, from medium-rank coals to anthracite.

### 3.6. Microscale Strength Properties Tests

**Figure 3:**
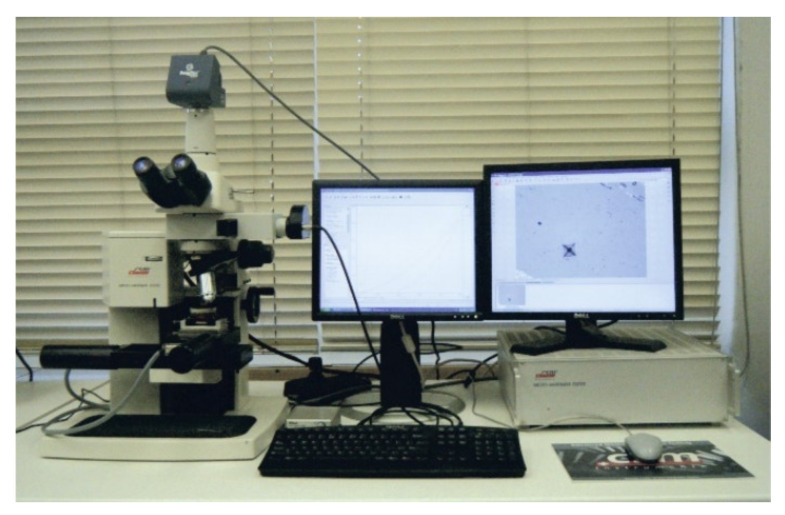
Stand for microhardness tests using the Vickers method.

A Micro Hardness Tester device by CSM Instruments, which includes a conical diamond Vickers indenter, an XY table and an image analysis software was used for the microhardness tests (Figure 3). Each measurement was followed by computer-assisted recording of experimental parameters and processing of the obtained results. The study included 15 to 20 measurements for every examined sample. The minimum, maximum and average values of the examined parameters were determined on this basis. The load used in coal analysis was 0.5 N.

The following parameters were examined:

– Vickers microhardness (*H_v_*), calculated according to the formula (4):
  (4) $$H_{v}=\frac{F_{max}}{9.81*A_{c}\left(h_{c}\right)}$$
  where:
  *F_max_*—maximum force (N).
  *h_c_*—indentation depth (μm).
  *A_c_*—surface area of the obtained indentation (μm^2^).
– standard modulus of elasticity (EIT), calculated according to the Power Law Method [49,69] from the formula (5):
  (5) $$E_{IT}=\frac{1-v_{s}^{2}}{\frac{1}{E_{r}}-\frac{1-v_{i}^{2}}{E_{i}}}$$
  where:
  *E_i_*—modulus of elasticity of the indenter—(constant, 1.141 GPa).
  *v_i_*—Poisson’s coefficient of the indenter (0.07).
  *E_r_*—reduced modulus of elasticity (which includes the influence of flexible deformation of the indenter under load).
  *ν_s_*—Poisson’s coefficient of the examined sample.
– the extent and nature of cracks and other damage occurring when the Vickers indenter is pressed against the examined material. This parameter is not described by any formulas, and its determination includes evaluation of surface deformation of the studied material by the observer. This may be used as the basis to evaluate such parameters as the brittleness of the examined material, since the sample returns to its initial position after the analysis, underneath the lens of an optical microscope, allowing observation and recording photographs of the location where the Vickers cone was pressed into the sample.

## 4. Results

### 4.1. Coal Rank and Sample Characteristics

The results of the technical analysis and of the vitrinite reflectance tests for the studied coals are presented in Table 1. As shown in Table 1, the vitrinite reflectance R_o_ for the studied coal samples ranged from 0.98% for the 404/4 F sample to 1.25% for the 502/1 E sample. The measured reflectance encompassed the characteristic values of medium-rank coal hard coal—types B and C (ortho and meta bituminous coals) according the UN-ECE classification [70]. According to van Krevelen [45], coal with a degree of coalification (R_o_) ranging from 0.98 to 1.25% contains between 86 and 87% of elemental C. The coal rank of the examined samples usually shows an increasing trend with increasing age of individual seams. The studied coals varied in terms of volatile content (13.82%$\;\leq V^{daf}\leq \;$29.08%) and ash content (2.58% $\leq \;A^{a}\leq \;$21.61%). showing relatively low moisture levels (0.82% ≤ $W^{t}$ ≤ 2.09%). The samples also showed variable porosity (1.2 ≤ ε ≤ 10%).

### 4.2. Sorption Test Results

**Figure 4:**
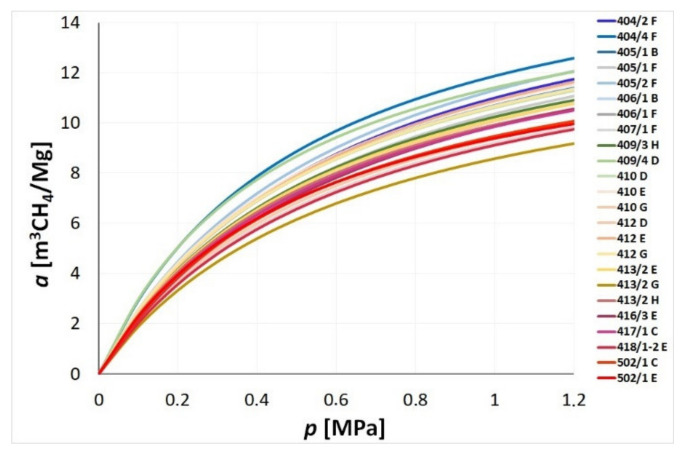
Methane sorption isotherms determined for the studied coals at 35 °C.

Figure 4 presents the results of the gravimetric sorption analysis performed at 35 °C in form of the sorption isotherms. Table 2 includes the values of $a_{m}$ and $p_{L}$ for the respective isothermal curves, obtained by approximation of sorption data using the Langmuir’s function (1). Values of the effective diffusion coefficient $D_{e}$, determined for individual coal samples according to the formula (3), are included in the last column in Table 2.

By comparing the sorption capacities summarised in Table 2, it can be observed that in the maximum sorption range (the *a_m_* value), the studied coals show a methane accumulation capacity between the value for the 413/2 G coal sample (14.15 m^3^CH_4_/Mg), and the quantity absorbed by the 405/2 F coal sample (18.25 m^3^CH_4_/Mg). Therefore, the course of methane sorption isotherms is slightly varied and the curves may intersect in some cases. As it may be seen from Figure 4, the isotherms more steeply inclined in the low-pressure range (lower value of the *p_L_* parameter) may quickly reach a plateau (e.g., sample 409/4 D), while a different curve, less inclined in the low pressure area, may have a much higher maximum sorption capacity $a_{m}$ (e.g., sample 405/2 F).

### 4.3. Microhardness

Results of the microhardness analysis performed for 24 coal samples from the Zofiówka area are presented in Table 3.

The next part of this paper presents an analysis of the influence of the degree of coalification on sorption properties (accumulation and kinetic properties) of coal seams towards methane and their microscale strength properties.

## 5. Discussion

### 5.1. Influence of the Degree of Coalification on Accumulation Sorption Properties

**Figure 5:**
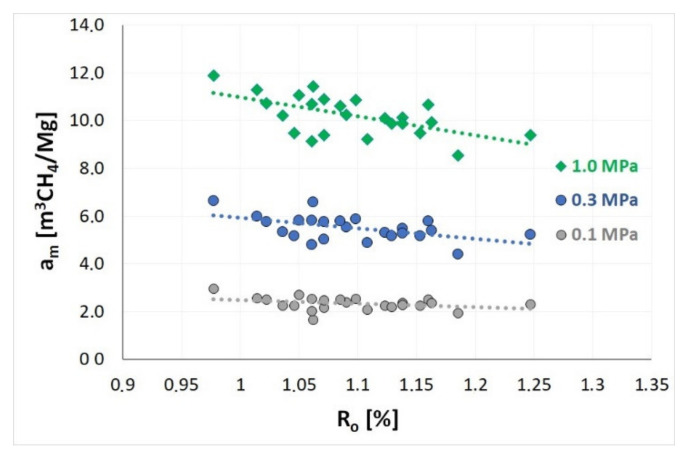
The influence of coalification degree on the sorption capacity (*a*) of coal under the given methane pressure.

**Figure 6:**
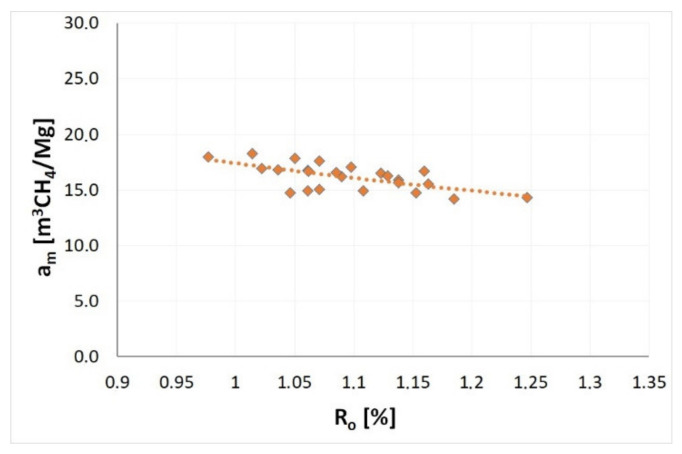
The impact of coalification degree on the maximum methane sorption capacity of coal (*a_m_*).

According to numerous literature sources, the degree of coalification, determined, e.g., on the basis of vitrinite reflectance, is the decisive key parameter for sorption properties of coal [26,71,72,73]. Figure 5 and Figure 6 present the relationships enabling the evaluation of the influence of the degree of coalification of coals prone to outburst on the sorption capacity of coal *a* under a given pressure—successively: 0.1 MPa, 0.3 MPa and 1.0 MPa, and on the maximum sorption capacity *a_m_*.

According to Figure 5 and Table 2, the values of the sorption capacity at the laboratory temperature for coal from various seams (0.98% < $R_{0}$ < 1.25%) vary within the following range: from 1.64 to 2.94 m^3^CH_4_/Mg at 0.1 MPa, from 4.39 to 6.64 m^3^CH_4_/Mg at 0.3 MPa and from 8.55 to 11.89 m^3^CH_4_/Mg at 1.0 MPa. As evident from Figure 5, the increased degree of coalification of the seams results in a systematic, approximately linear decrease of the sorption capacity of coal under the given methane pressure. Based on these trends, it can be determined that a reduction of the sorption capacity of coal with increasing degree of coalification of the seams between 0.98% and 1.25% is approximately 20%, regardless of the value of equilibrium pressure.

According to Figure 6 and Table 2, the maximum methane sorption capacity of coal for seams with various degrees of coalification varied in the range from 14.15 to 18.25 m^3^CH_4_/Mg at 35 °C. The relationships between the coal rank of samples and the maximum sorption capacity is also of a decreasing nature, which means a reduction of *a_m_* with increasing coalification (Figure 6). The relative change of the maximum sorption capacity resulting from the coalification of the seams increasing from 0.98% to 1.25% was approximately 18.5% at 35 °C. At a maintained constant seam temperature, the decreasing sorption capacity of coal progressed from younger seams with a lower degree of coalification towards older seams with a higher degree of coalification. In the ideal case, with the examined area of the orogeny lacking tectonic disturbances and the rock layers lying according to the lithostratigraphic profile, it could be stated that the accumulation properties of outburst coals deteriorate with the increasing depth at which the seam is located. The aforementioned observations coincide with observations included in works by other researchers, such as Laxminarayana and Crosdale [26] and Levine [74].

**Figure 7:**
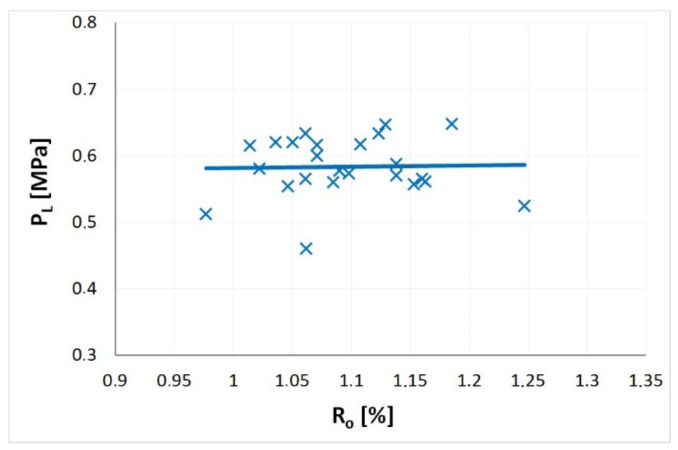
The influence of coalification degree on the Langmuir half sorption pressure (*P_L_*).

Figure 7 shows the influence of the degree of coalification on the value of Langmuir half sorption pressure $p_{L}$. In the range of the examined reflectances, from 0.98% to 1.25%, the coal rank change does not impact the mean value of Langmuir pressure (Figure 7). Most of the results are in the range of 0.55–0.65 MPa. However. some oscillations of the $p_{L}$ around the mean value can be observed. An analysis of literature data does not yield clear answers as to the variability of the Langmuir’s pressure as a function of the coal rank. Laxminarayana and Crosdale [26] showed that a clear decreasing relationship between the degree of coalification and the Langmuir pressure exists in the R_o_ reflectance range from 0.78% to 3.01%. A similar trend is presented in a work by Dutta et al. [73] for the R_o_ range from 0.6% to 1.9%. Some works also indicate the presence of an inverse relationship, which means that the Langmuir pressure increases with the increasing degree of coalification in the R_o_ range from approximately 0.25% or 1.4% (i.e., Lama and Bodziony [17]).

In the case of the examined coals with susceptibility to outburst, the increasing degree of coalification contributes to the lower sorption capacity of seams; however, the half sorption pressure remains virtually unchanged. Reduction of the methane sorption capacity of coal is clearly a warning sign to advise caution with respect to outburst activity.

### 5.2. Coal Seam Methane Pressure

Coal seam methane pressure is one of the key parameters important in the assessment of the state of outburst hazard and its value results from the sorption capacity of coal. Assuming the mean deposit temperature of 35 °C, the coal seam methane pressure was evaluated by performing a gas balance for these seams, according to the laboratory method described in Dutka and Godyń [25]. The calculations include values of Langmuir isotherm parameters $a_{m}$ and b (Table 2) and the respective coal porosities (Table 1). The coal seam methane pressures were calculated assuming the total methane content criterion of *Mn* = 8 m^3^CH_4_/Mg [12].

**Figure 8:**
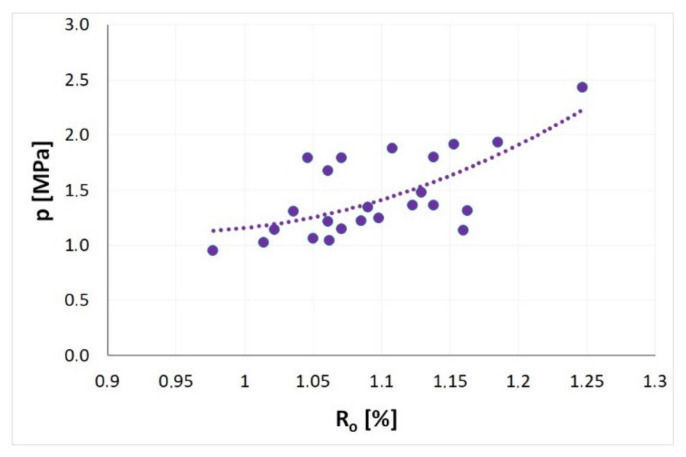
Impact of coalification degree on the seam methane pressure (*Mn* = 8 m^3^CH_4_/Mg, 35 °C).

Figure 8 presents the influence of the degree of coalification on the coal seam methane pressure. According to the trend presented in Figure 8, the increase of the *p* value was approximately 3.3 MPa for every 1% increase of R_o_. This means that in the case of the examined coals, a 0.1% increase of the degree of coalification results in an average increase of the coal seam methane pressure of approximately 0.33 MPa. The increasing trend of the coal seam methane pressure is caused mainly by the sorption properties of the seams, and in particular by the sorption capacity, which is reduced with increasing degree of coalification in the case of the examined coals. At constant total methane content, coals with stronger absorption performance (with lower R_o_ values) require lower values of the methane equilibrium pressure, in contrast with coals exhibiting weaker sorption properties (with higher coal ranks). Porosity is the second important factor influencing the division of seams into two groups. Coals with low and very low porosity require higher seam pressures in order to reach the methane balance for the seam at the level of *Mn* = 8 m^3^CH_4_/Mg.

Increasing coal seam methane pressure is always an undesirable factor, as it indicates an increased risk of outburst. Even though literature data does not specify a threshold value for the coal seam methane pressure at which an outburst can occur, an increasing value should be a warning sign for the mine personnel with regard to a potential risk.

With mining operations going deeper, and therefore reaching for more coalified seams, reduction of the methane sorption capacity of coal seams and their porosity are a particularly unfavourable phenomenon from the point of view of gas-geodynamic processes.

### 5.3. Desorbable Methane Content

**Figure 9:**
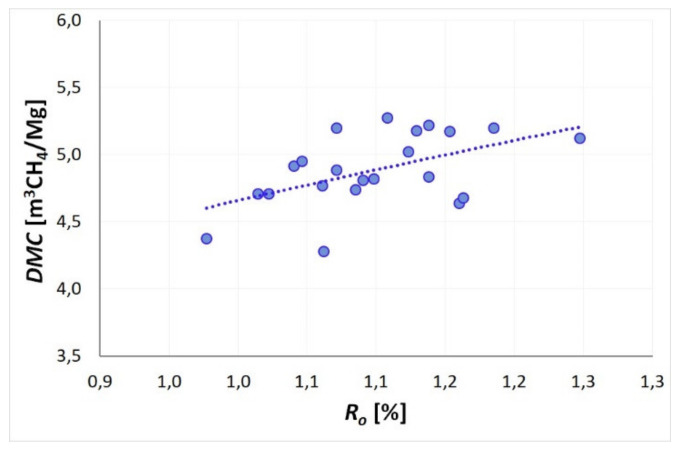
Desorbable methane content as a function of coalification degree (*Mn* = 8 m^3^CH_4_/Mg, 35 °C).

In order to determine the desorbable methane content *DMC*, the total methane content *Mn* was decreased for each of the seams by sorption capacity *a*_0.1MPa_ under the atmospheric pressure (see Table 1) and free methane w, present in the pore volume of the coal. A constant value of total methane content (8m^3^CH_4_/Mg) and a mean deposit temperature of 35 °C were assumed. Figure 9 presents the variability of desorbable methane content related to changes in the degree of coalification of the examined seams. According to the presented graph, an increase in the degree of coalification of the seams results in an increase of the fraction of desorbable methane *DMC* in the total methane content. Therefore, with the increasing depth of seams deposition, the potential quantity of methane released into the excavation during a coal and methane outburst is increased. Similarly, the tendency presented in Figure 9 is related to the results of sorption test presented in Section 5.1. At a specified value of *Mn*, poorly absorbing coals (with a higher coal rank) show a lower sorption capacity *a*_0.1MPa_ resulting in the observed increase of desorbable methane content *DMC*. Therefore, the increasing degree of coalification of coal seams increases the amount of gas potentially released from the seam disturbed by mining activities and the gas-geodynamic potential of coal seams located deeper underground.

### 5.4. Influence of the Degree of Coalification on Kinetic Sorption Properties

**Figure 10:**
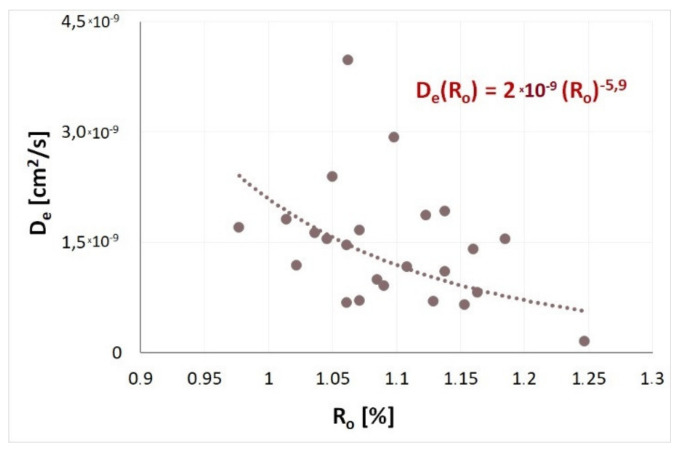
Influence of the degree of coalification on the effective diffusion coefficient.

Table 2 presents the half-times $t_{0.5}$ and values of the effective diffusion coefficient $D_{e}$ determined for the examined coals for a fill level of *γ* = 0.5. According to Table 2, sorption half-times vary at 35 °C between 390 s for sample 409/4 D and 10^4^ s for sample 502/1 E. Values of the effective diffusion coefficient $D_{e}$ corresponding to the listed half-times, cover a variability range from 1.55 × 10^−10^ cm^2^/s to 3.98 × 10^−8^ cm^2^/s, and the relatively high variability of the effective diffusion coefficient, covering two orders of magnitude, shows that coals susceptible to outburst are characterised by a different internal structure resulting from changes in the degree of coalification. The influence of the degree of coalification on the kinetic properties of methane sorption is shown in Figure 10.

Within the examined reflectance range R_o_, the effective diffusion coefficient of methane decreased with the increasing degree of coalification of the samples. The decreasing methane sorption rate, coinciding with the power trend, was present up to a certain, asymptotic value of *D_e_*. Figure 10 shows that sorption and diffusion processes of coals with outburst susceptibility are slower in seams with a higher coal rank than in seams with a lower coal rank. Therefore, natural coalification results in the ordering of structure and an increased size of coal micro- and sub-microporosity, as stated, for example by Zarębska and Dudzińska [75]. These changes make gas penetration into the coal structure more difficult.

**Figure 11:**
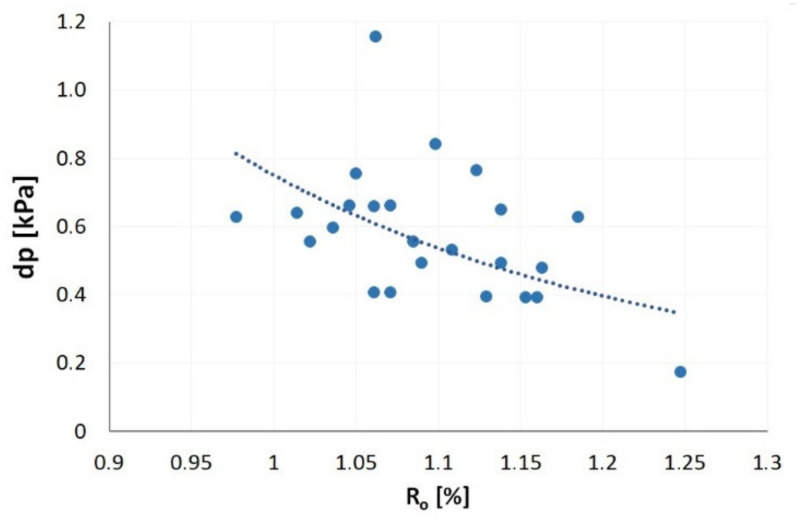
The influence of coalification degree on the value of desorption intensity index.

Knowing the model for methane release from coal [66,67], the value of the effective diffusion coefficient at a given temperature (Table 2) and the grain class desorption intensity indexes were modelled. In the case of the polish desorbometer, emission of 1 cm^3^ of the gas causes a change of the liquid level corresponding to the desorption index dp of 1.73 kPa [76]. If the geometric dimensions of the desorbometer are known and assuming constant total methane content (*Mn* = 8 m^3^CH_4_/Mg), the *dp* values were calculated for the studied coals and the respective *D_e_* values (Figure 11). The desorption intensity index dp of coals susceptible to outburst varied from 0.17 kPa to 1.16 kPa and the tendency of *dp* versus R_o_ was similar to that shown in Figure 10.

Higher values of *dp* index may occur in coal seams of higher diffusivity, assuming similar total methane content of coal *Mn*. It should be noted that increase in the total methane content of the seam also contributes to the increase of desorption intensity index *dp* [76]. Thus, as a result of an increase in coalification degree and reduction of methane emission rate (Figure 10), a higher desorbometer reading is an important warning sign indicating an increased total methane content coal seam or an alternation of coal structure (disturbed coal). The increased *dp* also indicates a higher value of seam methane pressure.

### 5.5. Microhardness Properties and the Degree of Coalification

**Figure 12:**
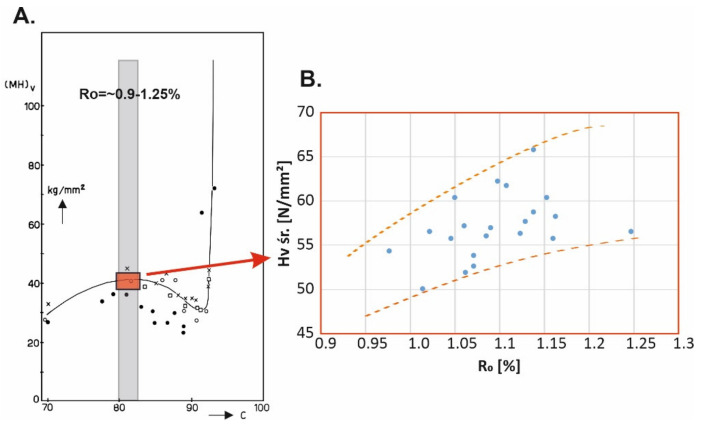
(**A**) Microhardness as a function of coalification of organic matter (according to Stach [28]); (**B**) Microhardness as a function of vitrinite reflectance of the studied coal samples.

Literature data shows that microhardness (H_v_) depends on the degree of coalification. Figure 12A presents a graph showing the relationship between Vickers microhardness and the degree of coalification of organic matter [28]. The range of the degree of coalification for the examined coal samples is presented in the graph. Within the marked area along according to the plotted line, microhardness should either increase slightly or remain constant with increasing degree of coalification. It can therefore be concluded that with increasing R_o_, the examined samples should have similar microhardness or display a slight H_v_ increase with increasing degree of coalification.

The graph (Figure 12B) shows a certain relationship between R_o_ and H_v_ in conformity with literature data. A small H_v_ increase is observed with increasing degree of coalification.

In comparison with other coals described in the literature with different coal ranks the examined samples show specific characteristics also defining their properties in the context of broadly understood tendency and proneness to the occurrence of potential gas-geodynamic hazards. According to literature data, low-rank coal containing approximately 70% elemental C (corresponding to R_o_ = ~0.2% (Stach et al. [28]; van Krevelen [29]) is a coal with relatively low brittleness and hardness in which the indentation of the Vickers cone is very weak or barely visible. The cone indentation is clearly visible in a medium-rank coal with increasing microhardness and occurrence of brittle deformations. After Stach et al. [28], coal microhardness increases up to approx. 88% elemental C (or R_o_ > 1.2%) and this value marks the end of the range of brittle vitrinite fracture. In coal with a content of 88 to 92% elemental C (R_o_ in the range from ~1.25 to ~2.7%) microhardness decreases as vitrinite enters the range of plastic deformations. Microhardness clearly increases in coal containing more than 92% elemental C (anthracite) and vitrinite becomes elastic. In anthracite, the effect of the Vickers cone visible in the indentation is similar to very low-rank coals. however. there is a notable difference in their microhardness [28]. Studies conducted in recent years (own and of other researchers) proves that the results of coal microhardness parameter analysis with the changing degree of coalification may be somewhat different than the previous literature data (data from [30]). However, all study results, both those included in the archival studies and the current ones, presented recently by the authors of this publication show that microhardness properties may be an sign of specific coal behaviour and indicate features responsible for increased coal outburst susceptibility.

Results contained in the study [23,30] were used to compare and analyse the behaviour of coal and of its properties with increasing coal ranks. Owing to the results available for a group of low rank samples, with R_o_ of about 0.8% and for high rank samples with R_o_ of approximately 2.0%, different properties of coal with different coal ranks were shown (Table 4).

The coal rank determines the cracking of the examined vitrinite fragments. The nature of the changes occurring once the Vickers pyramid is pressed into coal fragments provides the information that indicates whether the given substance is able to accumulate energy or exhibits plasticity [47]. The coal in which brittle deformations (cracks, crevices, detached surface fragments) occur when a specific pressure is able to accumulate a certain amount of energy which is released in the form of brittle deformations upon breaking by a specific force. If after application of the Vickers indenter no other surface deformations apart from the cone or cross are not observed. it may be assumed that this substance is undergoing plastic or elastic deformation. According to the characteristics of the described coal, its parts made of vitrinite show a tendency to brittleness. This type of deformation indicates the behaviour of coal in the presence of a fault or when a certain force is applied, e.g., as a result of mining works. Such material, containing accumulated energy, may suddenly and uncontrollably break facilitating gas-geodynamic phenomena if gas is involved. Therefore, material properties within a microarea may describe the coal behaviour.

The way vitrinite fractures after the performed examinations in almost all samples shows that they are capable of brittle cracking. Therefore, the coal has the ability to accumulate energy. Cracks and certain defects present in some grains are a result occurring after the Vickers microhardness tests, however, their form does not disturb the depression formed in vitrinite after the application of the Vickers cone.

## 6. Conclusions

The degree of coalification is responsible for sorption properties and for strength properties of coal. measured on the micro scale. The susceptibility of coals to outbursts is also closely related to the coalification of seams, which contributes to undesirable properties of seams increasing the probability of outbursts in the conditions of increasing total methane content, decreasing sorption capacity and coal diffusivity. The information on the variability of the sorption capacity of coal and of the effective diffusion coefficient is an important factor for forecasting gas-geodynamic phenomena in active mines in view of the availability of the south-western part of the USCB.

The consequences of the variability of sorption properties of coal as a function of increasing degree of coalification described in this paper include primarily:

- increase of the equilibrium pressure of methane in highly coalified seams;
- increase of the desorbable methane content in the total methane content;
- a reduction in the measured methane desorption intensity indices from coal during work that made the seam available.

The properties of coal displayed in microhardness properties clearly show that microhardness determined using the Vickers method allows the behaviour of coal material under the applied force to be observed. The material undergoes deformations and shows different behaviour, depending on the coalification of organic matter. The presented analyses show that medium-rank coal, namely coal from the Zofiówka Mine area examined in the present paper, is the most brittle, and therefore most prone to outbursts. This area is particularly susceptible to uncontrollable gas-geodynamic phenomena in correlation with the data showing that methane and numerous faults formed over geological time scales are present in the seams in this area.

As evident from this study, a compilation of capacity and kinetic sorption parameters, combined with information about the mechanical parameters (including the micro scale) enables assessment of the possible outburst risk in mines. Laboratory tests effectively support the assessment of coal and methane outburst hazards.

The conducted research concerned coal seams with medium degree of coalification, which were susceptible to outbursts. It was an introduction to a broader study of coals in a wide range of coalification degree. Further research will make it possible to emphasise the features of the outburst coals in comparison to other coalification degrees and their measurable dissimilarity, which contributes to the outburst’s occurrence.

## Figures and Tables

**Table 1:** Designation of coal samples, place of sampling, average vitrinite reflectance and technical analysis.

Sample	Part	Seam	Lithostratigraphy	Formation	Age	*R*_0_, %	*V^daf^*, %	*A^d^*, %	*W^t^*, %
404/2 F	F	404/2	załęskie	Siltstone	Westphalian A	1.050	25.29	5.01	2.09
404/4 F	F	404/4	załęskie			0.977	24.78	6.00	1.84
405/1 B	B	405/1	załęskie			1.022	28.45	10.19	1.36
405/1 F	F	405/1	załęskie			1.036	20.58	11.57	1.19
405/2 F	F	405/2	załęskie			1.014	22.33	15.68	1.16
406/1 B	B	406/1	załęskie			1.061	20.92	2.69	1.59
406/1 F	F	406/1	załęskie			1.046	27.84	2.58	1.95
407/1 F	F	407/1	rudzkie	Upper Silesian Sandstone	Namurian C	1.108	23.25	6.31	1.13
409/3 H	H	409/3	rudzkie			1.090	19.89	6.73	1.28
409/4 D	D	409/4	rudzkie			1.062	29.08	20.20	1.12
410 D	D	410	rudzkie			1.085	17.81	8.78	1.44
410 E	E	410	rudzkie			1.098	20.79	7.54	1.57
410 G	G	410	rudzkie			1.071	20.20	5.56	0.88
412 D	D	412	rudzkie			1.123	19.34	21.61	0.82
412 E	E	412	rudzkie			1.071	19.42	11.77	1.39
412 G	G	412	rudzkie			1.160	17.40	4.38	1.06
413/2 E	E	413/2	rudzkie			1.138	18.35	3.49	1.31
413/2 G	G	413/2	rudzkie			1.185	18.71	4.28	1.62
413/2 H	H	413/2	rudzkie			1.163	25.01	10.27	0.92
416/3 E	E	416/3	rudzkie			1.129	19.17	9.57	1.00
417/1 C	C	417/1	rudzkie			1.138	18.33	12.25	1.39
418 E	E	418	rudzkie			1.061	16.54	8.02	1.32
502/1 C	C	502/1	siodłowe		Namurian B	1.153	21.44	8.76	1.08
502/1 E	E	502/1	siodłowe			1.247	13.82	6.48	1.15

$R_{0}$: vitrinite reflectance; $V^{daf}$: volatile matter yield with dry-ash-free basis; $A^{d}$: ash yield with dry basis; $W^{t}$: moisture content with air-dried basis.

**Table 2:** Results of sorption tests with methane at 35 °C, parameters of Langmuir isotherms *a_m_* and *p_L_*, sorption half-time *t*_0.5_ and values of the effective diffusion coefficient $D_{e}$.

*Sample*	*a_m_* [m^3^CH_4_/g]	*p_L_* [MPa]	*t*_0.5_ [s]	*D_e_* [cm^2^/s]
404/2 F	17.814	0.620	650	2.39 × 10^−9^
404/4 F	17.966	0.512	915	1.70 × 10^−9^
405/1 B	16.920	0.580	1310	1.19 × 10^−9^
405/1 F	16.801	0.620	950	1.63 × 10^−9^
405/2 F	18.254	0.615	860	1.81 × 10^−9^
406/1 B	16.751	0.565	1060	1.46 × 10^−9^
406/1 F	14.727	0.554	1000	1.55 × 10^−9^
407/1F	14.910	0.617	1330	1.17 × 10^−9^
409/3 H	16.183	0.577	1705	9.11 × 10^−10^
409/4 D	16.680	0.460	390	3.98 × 10^−9^
410 D	16.557	0.560	1570	9.89 × 10^−10^
410E	17.072	0.573	530	2.93 × 10^−9^
410 G	15.012	0.600	2200	7.06 × 10^−10^
412 D	16.492	0.633	831	1.87 × 10^−9^
412 E	17.600	0.616	930	1.67 × 10^−9^
412 G	16.680	0.565	1100	1.41 × 10^−9^
413/2 E	15.907	0.570	807	1.92 × 10^−9^
413/2 G	14.154	0.648	1000	1.55 × 10^−9^
413/2 H	15.512	0.561	1900	8.17 × 10^−10^
416/3 E	16.258	0.647	2220	6.99 × 10^−10^
417/1 C	15.649	0.587	1410	1.10 × 10^−9^
418 E	14.885	0.633	2270	6.84 × 10^−10^
502/1 C	14.750	0.557	2382	6.52 × 10^−10^
502/1 E	14.326	0.524	10,000	1.55 × 10^−10^

**Table 3:** Results of reflectance tests and microhardness properties for coal samples from the Zofiówka mine.

Coal Sample	H_v_ [N/mm^2^]	E_it_ [GPa]	H_v_ Max [N/mm2]	E_it_ Max [GPa]	H_v_ Min [N/mm^2^]	E_it_ Min [GPa]
404/4 F	54.32	5.70	61.00	6.17	42.83	4.94
405/2 F	50.04	5.65	64.49	6.44	40.40	5.01
405/1 B	56.51	5.79	64.77	6.38	46.72	5.27
406/1 F	55.71	6.00	66.40	6.63	44.03	5.02
404/2 F	60.37	5.30	74.96	6.23	35.12	3.99
406/1 B	57.18	6.07	67.95	6.84	41.76	4.89
418 E	64.44	6.46	76.59	7.19	47.80	5.62
409/4 D	51.89	5.58	62.34	6.21	43.57	5.02
410 G	53.86	5.95	62.52	6.66	43.47	5.25
412 E	52.59	5.65	64.19	6.36	43.37	5.05
410 D	56.07	5.94	69.86	6.77	46.62	5.22
409/3 H	56.96	6.19	71.63	7.04	42.56	5.30
410 E	62.2	6.05	76.25	6.66	58.29	5.74
407/1 F	61.75	6.15	69.79	6.91	51.30	5.17
412 D	56.3	5.98	75.52	6.87	44.24	4.94
416/3 E	57.67	5.99	65.63	6.68	44.42	5.31
413/2 E	58.71	6.04	73.44	6.84	41.94	5.04
417/1 C	65.75	6.46	73.00	7.07	46.72	5.21
502/1 C	60.39	5.98	73.85	6.47	45.04	5.25
412 G	55.75	5.88	68.56	6.19	41.82	5.07
413/2 H	58.21	6.63	65.86	7.14	44.29	5.75
413/2 G	47.43	5.63	67.20	6.83	39.97	4.98
502/1 E	56.51	5.79	64.77	6.38	46.72	5.27

R_o_—average vitrinite reflectance; H_v_—average Vickers microhardness; E_it_—average modulus of elasticity. H_v_ max—maximum Vickers microhardness; E_it_ max—maximum modulus of elasticity; H_v_ min—minimum Vickers microhardness; E_it_ min—minimum modulus of elasticity.

**Table 4:** Differences in microhardness of coals with different degrees of coalification.

	Low-Rank Coals (wg Godyń et al. [30])	Medium-Rank Coals	High-Rank Coals (wg Godyń et al. [30])
R_o_	0.78–0.85%	0.98–1.25%	1.85–2.03%
Average value of H_v_	69.25	57.42	57.11
Photographs after measurement	* 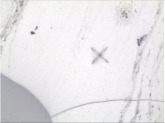 *	* 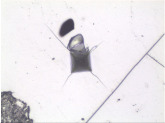 *	* 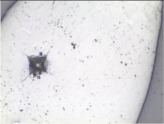 *
Notes	The coals have the highest microhardness and the Vickers cone leaves a small mark. indicating that low-rank coal does not show a tendency to break under the applied load. The samples are slightly deformed. showing a certain plasticity at a high value of (H_v_).	A significant decrease of microhardness was observed. The Vickers cone indentation leaves clear marks of the performed measurement. Extensive cracks running from the tops of the indentations were observed. sometimes accompanied by separated material fragments. The damage indicates that this material is crushed very easily.	A slight H_v_ decrease occurs. A Vickers pyramid trace is formed on the sample surface, and the cracks around the indentation are not as spectacular as in medium-rank samples. Cracks and separated fragments are visible. but most coal changes are focused in the indentation and nearby.

## Data Availability

The data used to support the findings of this study are available from the corresponding author upon request.

## References

[1] (2021). International Trade Administration. https://www.trade.gov/.

[2] Gawlik L. (2018). The Polish power industry in energy transformation process. Miner. Econ..

[3] Czerski G., Dziok T., Porada S. (2014). Możliwości wykorzystania technologii zgazowania węgla do wytwarzania energii, paliw i produktów chemicznych. Polityka Energetyczna.

[4] Klank M. (2007). Przyszłość węgla—Nowe spojrzenie na jego wykorzystanie. Polityka Energetyczna.

[5] Stala-Szlugaj K. (2016). Trends in the consumption of hard coal in Polish households compared to EU households. Gospod. Surowcami Miner..

[6] Kędzior S. (2009). Accumulation of coal-bed methane in the south-west part of the Upper Silesian Coal Basin (southern Poland). Int. J. Coal Geol..

[7] Dutka B. (2021). Effect of depth on the sorption capacity of coals affected by outburst hazard. Fuel.

[8] Beamish B., Crosdale P.J. (1998). Instantaneous outbursts in underground coal mines: An overview and association with coal type. Int. J. Coal Geol..

[9] Wierzbicki M., Młynarczuk M. (2006). Microscopic analysis of structure of coal samples collected after an gas and coal outbursts in the gallery D-6, coal seam 409/4 in the “Zofiówka” coal mine (upper silesian coal basin). Arch. Min. Sci..

[10] Wierzbicki M., Jakubów A., Tor A. (2012). Wyrzut Metanu i Skał w Pochylni D Odstawczej W Pokładzie 358/1 Na Poziomie 1050 M W JSW S.A. KWK “Budryk”—Przyczyny: Okoliczności Skutki.

[11] Brook N., Misra B. A Critical Analysis of the Stamp Mill Method of Determining Protodyakonov Rock Strength and the Development of a Method of Determining a Rock Impact Hardness Number. Proceedings of the 12th U.S. Symposium on Rock Mechanics.

[12] Skoczylas N., Wierzbicki M. (2014). Evaluation and management of the gas and rock outburst hazard in the light of international legal regulations. Arch. Min. Sci..

[13] Lama R.D., Bodziony J. (1998). Management of outburst in underground coal mines. Int. J. Coal Geol..

[14] Godyń K. (2012). The effect of tectonic discontinuities upon the internal structure of coal from some Upper Silesian Coal Basin coal seams of Pniówek, Borynia-Zofiówka and Brzeszcze. Biul. Państwowego Inst. Geol..

[15] Godyń K. (2013). Characteristics of hard coal in the near-fault zones. Przegląd Górniczy.

[16] Godyń K. (2016). Structurally altered hard coal in the areas of tectonic disturbances—An initial attempt at classification. Arch. Min. Sci..

[17] Lama R.D., Bodziony J. (1996). Outbursts of Gas, Coal and Rock in Underground Coal Mines.

[18] Shepherd J., Rixon L.K., Creasey J.W. (1980). Analysis and Prediction of Geological Structures Associated with Outbursts at Collinsville, Queensland.

[19] Cao Y., Mitchell G.D., Davis A., Wang D. (2000). Deformation metamorphism of bituminous and anthracite coals from China. Int. J. Coal Geol..

[20] Li H., Ogawa Y., Shimada S. (2003). Mechanism of methane flow through sheared coals and its role in methane recovery. Fuel.

[21] Skiba M. (2016). The influence of the discrepancies in the observers’ decisions on the process of identification of maceral groups using artificial neural networks. J. Sustain. Min..

[22] Skiba M., Młynarczuk M. (2020). Estimation of coal’s sorption parameters using artificial neural networks. Materials.

[23] Godyń K., Kožušníková A. (2019). Microhardness of coal from near-fault zones in coal seams threatened with gas-geodynamic phenomena, Upper Silesian Coal Basin, Poland. Energies.

[24] Godyń K., Dutka B. (2018). The impact of the degree of coalification on the sorption capacity of coals from the Zofiówka Monocline. Arch. Min. Sci..

[25] Dutka B., Godyń K. (2018). Predicting variability of methane pressure with depth of coal seam. Przemysł Chem..

[26] Laxminarayana C., Crosdale P. (1999). Role of coal type and rank on methane sorption characteristics of Bowen Basin, Australia coals. Int. J. Coal Geol..

[27] Manecki A., Muszyński M. (2008). Przewodnik do Petrografii.

[28] Stach E., Mackowsky M.-T., Teichmuller M., Taylor G.H., Chandra D., Teichmuller R. (1982). Stach’s Textbook of Coal Petrology.

[29] Van Krevelen D.W., Schuyer J. (1959). Węgiel. Chemia węgla i jego struktura.

[30] Godyń K., Dutka B., Chuchro M., Młynarczuk M. (2020). Synergy of parameters determining the optimal properties of coal as a natural sorbent. Energies.

[31] Gray I. (1987). Reservoir engineering in coal seams: Part. 1—The physical process of gas storage and movement in coal seams. SPE Reserv. Eng..

[32] Moore T.A. (2012). Coalbed methane: A review. Int. J. Coal Geol..

[33] Dutka B. (2019). CO_2_ and CH_4_ sorption properties of granular coal briquettes under in situ states. Fuel.

[34] Skoczylas N. (2015). Determining the gas permeability coefficient of a porous medium by means of the bubble-counting flow meter. Meas. Sci. Technol..

[35] Skoczylas N. (2015). Analyzing the parameters of the coal—Gas system using a low-cost device based on a flowmeter. Adsorpt. Sci. Technol..

[36] Skoczylas N., Topolnicki J. (2016). The coal-gas system—The effective diffusion coefficient. Int. J. Oil Gas Coal Technol..

[37] Charrière D., Pokryszka Z., Behra P. (2010). Effect of pressure and temperature on diffusion of CO_2_ and CH_4_ into coal from the Lorraine basin (France). Int. J. Coal Geol..

[38] Wierzbicki M. (2013). Changes in the sorption/diffusion kinetics of a coal-methane system caused by different temperatures and pressures. Gospod. Surowcami Miner..

[39] Xu H., Tang D., Zhao J., Li S., Tao S. (2015). A new laboratory method for accurate measurement of the methane diffusion coefficient and its influencing factors in the coal matrix. Fuel.

[40] Wierzbicki M., Dutka B. (2010). The influence of temperature changes of the structurally deformed coal—Methane system on the total methane content. Arch. Min. Sci..

[41] Mukherjee A.K., Alam M.M., Ghose S. (1989). Microhardness characteristics of Indian coal and lignite. Fuel.

[42] Tora B., Fecko P., Nowak A., Tajchman Z. (2010). Investigation on dependence of grindability on chosen parameters of hard coal. Górnictwo Geoinżynieria.

[43] Yuzo S., Hidemasa H. (1962). Microhardness of coal immersed in various solvents. Bull. Chem. Soc. Jpn..

[44] Eremin I.V., Lebedev V.V., Cikarev A.D. (1980). Petrografija i Fizičeskije Svojstva Uhlej.

[45] Van Krevelen D.W. (1993). Coal–Typology–Physics–Chemistry–Constitution.

[46] Beneš K. (1957). Měření mikrotvrdosti uhelně-petrografických složek a mikrolitotypů Hanemanovým mikrotvrdoměrem. Sborník Vědeckých Prací Vysoké Školy Báňské.

[47] Das B. (1968). Microhardness Study of Coal with Special Reference to Mechanism of Dust Genesis. Master’s Thesis.

[48] Martinec P., Kožušníková A. Mikrotvrdost černých uhlí ostravsko-karvinského revíru–dosa-vadní stav a možnosti dalšího rozvoje problematiky. Documenta Geonica. Proceedings of the 6th Czech-Polish Conference of the Upper Silesian Coal Basin.

[49] Kožušníková A. (2009). Determination of microhardness and elastic modulus of coal components by using indentation method. Geolines.

[50] Bukowska M., Ćmiel S. (2011). The characteristic of changes of Carboniferous rocks properties in non-continuous tectonic zones in the Upper Silesian Coal Basin. Górnictwo Geoinżynieria.

[51] Ćmiel S.R. (2009). Charakterystyka Epigenetycznych Zmian Węgla w Pokładach w Strefach Uskokowych Górnośląskiego Zagłębia Węglowego.

[52] Godyń K., Kralova L. (2017). Application of Vickers micro hardness measurements for hard coal analysis focusing on the F part of the Borynia–Zofiówka–Jastrzębie mine, Zofiówka Section. Trans. Strata Mech. Res. Inst..

[53] Hower J.C., Trinkle E.J., Raione R.P. (2008). Vickers microhardness of telovitrinite and pseudovitrinite from high volatile bituminous Kentucky coals. Int. J. Coal Geol..

[54] Given P.H. (1976). The use of DAF and DMMF ultimate analyses of coals. Fuel.

[55] Kus J., Misz-Kennan M. (2017). Coal weathering and laboratory (artificial) coal oxidation. Int. J. Coal Geol..

[56] Markova K., Valceva S. (1983). Oxidation of some Bulgarian coals. Influence of low-temperature oxidation on the microhardness and reflectivity of some brown coals. Fuel.

[57] Nandi B.N., Ciavaglia L.A., Montgomery D.S. (1977). The variation of the microhardness and reflectance of coal under conditions of oxidation simulating weathering. J. Microsc..

[58] Gabzdyl W. (1994). Geologia Złóż Węgla: Złoża Świata.

[59] Osika R. (1987). Budowa Geologiczna Polski, Tom VI; Złoża Surowców Mineralnych.

[60] Kotas A. (1994). Coalbed methane potential of the Upper Silesian Coal Basin, Poland.

[61] Probierz K., Marcisz M., Sobolewski A. (2012). Od Torfu do Węgli Koksowych Monokliny Zofiówki w Obszarze Jastrzębia (Południowo-Zachodnia Część Górnośląskiego Zagłębia Węglowego).

[62] Praca zbiorowa pod kierunkiem dr. (2019). hab. inż. Józefa Kabiesza. Report on the State of Basic Natural and Technical Hazards in Hard Coal Mining in 2018.

[63] The Polish Geological Institute—National Research Institute. http://geoportal.pgi.gov.pl.

[64] Dutka B., Godyń K. (2021). Coalification as a process determining the methane adsorption ability of coal seams. Arch. Mining Sci..

[65] Klika Z., Serenčíšová J., Kožušníková A., Kolomazník I., Študentová S., Vontorová J. (2014). Multivariate statistical assessment of coal properties. Fuel Process. Technol..

[66] Timofejew D.P. (1967). Adsorptionskinetik.

[67] Crank J. (1975). The Mathematics of Diffusion.

[68] Busch A., Gensterblum Y. (2011). CBM and CO2-ECBM related sorption processes in coal: A review. Int. J. Coal Geol..

[69] Oliver W.C., Pharr G.M. (1992). An improved technique for determining hardness and elastic modulus using loadand displacement sensing indentation experiments. J. Mater. Res..

[70] UN-ECE (1998). International Classification of In-Seam Coals.

[71] Levy J.H., Day S.J., Killingley J.S. (1997). Methane capacities of Bowen Basin coals related to coal properties. Fuel.

[72] Prinz D., Pyckhout-Hintzen W., Littke R. (2004). Development of the meso and macroporous structure of coals with rank as analyzed with small angle neutron scattering and adsorption experiments. Fuel.

[73] Dutta P., Bhowmik S., Das S. (2011). Methane and carbon dioxide sorption on a set of coals from India. Int. J. Coal Geol..

[74] Levine J.R., Law B.E., Rice D.D. (1993). Coalification: The Evolution of Coal as Source Rock and Reservoir.

[75] Zarębska K., Dudzińska A. (2008). Możliwości Magazynowania CO_2_ w Pokładach Węgli Kamiennych—Weryfikacja Danych Eksperymentalnych.

[76] Wierzbicki M. (2011). Effect of selected simplifications of the unipore model upon the result of the study of the diffusion coefficient. Arch. Min. Sci..

